# Eye diseases and dementia: association because of pathogenesis or visual impairment?

**DOI:** 10.1002/alz70860_099176

**Published:** 2025-12-23

**Authors:** Xinyu Gu, Pauline Terebuh, Rong Xu, David Kaelber, Pamela Davis

**Affiliations:** ^1^ Case Western Reserve University School of Medicine ‐ ‐ Cleveland, OH, Cleveland, OH, USA; ^2^ Case Western Reserve University, Cleveland, OH, USA; ^3^ Case Western Reserve University School of Medicine, Cleveland, OH, USA

## Abstract

**Background:**

Research suggests a link between blindness, age‐related macular degeneration (AMD), and a higher risk of dementia. Since AMD can lead to blindness, it remains unclear whether this elevated risk is due to the disease's underlying pathology or its ability to cause blindness. The presence of Aβ amyloid proteins in AMD retinas’ drusen adds further complexity. This study aims to determine whether the dementia risk is attributed to AMD pathogenesis or the resulting blindness.

**Method:**

We conducted a retrospective cohort study using TriNetX, a deidentified electronic health record platform, focusing on patients aged over 50 years. Non‐blind patients with exudative AMD (*n* = 36,138) and non‐exudative AMD (*n* = 102,494) were compared to non‐blind individuals without AMD (*n* = 1,901,246). We calculated hazard ratios (HR) for Alzheimer's disease (AD), vascular dementia (VaD), and all‐cause dementia over five years. Additionally, we compared blind patients (*n* = 59,835) with non‐blind patients (*n* = 920,118) for the same outcomes. Cohorts were propensity‐matched for age, gender, race/ethnicity, and other dementia risk factors. Sensitivity analyses assessed dementia risk across age groups and by AMD status in blind patients.

**Result:**

AMD in non‐blind patients was either not associated with dementia or linked to a reduced risk, while blindness showed a strong positive association. Exudative AMD showed no increased VaD risk (HR=0.825, CI = [0.68, 1.001]) but had a significantly reduced risk of AD (HR=0.815, CI=[0.697, 0.952]) and all‐cause dementia (HR=0.792, CI=[0.766, 0.818]). Non‐exudative AMD also showed no significant risk of VaD (HR=0.893, CI=[0.791, 1.009]) but had a significantly reduced risk of AD (HR=0.86, CI = [0.782, 0.947]) and all‐cause dementia (HR=0.862, CI=[0.818, 0.908]). Blindness was linked to a significantly increased risk of AD (HR=1.429, CI = [1.276, 1.602]), VaD (HR=1.665, CI=[1.488, 1.862]), and all‐cause dementia (HR=1.383, CI=[1.304, 1.466]). This pattern is consistent across various age groups in patients above 50 years old and in blind patients as well.

**Conclusion:**

AMD is associated with a mildly reduced risk of Alzheimer's dementia when controlling for blindness. Prior reports linking AMD to dementia likely reflect the impact of blindness rather than shared pathology. This protective effect may be due to AMD treatments like AREDS2 vitamins, which support cognitive health.

